# Play in School – Toward an Ecosystemic Understanding and Perspective

**DOI:** 10.3389/fpsyg.2021.780681

**Published:** 2022-01-28

**Authors:** Helle Marie Skovbjerg, Anne-Lene Sand

**Affiliations:** Lab for Play and Design, Design School Kolding, Kolding, Denmark

**Keywords:** play, children, education, play theory, pedagogy

## Abstract

Based on a design-based research project and long-term observations of children’s play in school, this article develops the concept of *play order*, which points to interaction, coherence and holistic orientation as central values for the approach to play in school. Through concrete empirical analysis, the article shows how play in school is established and maintained, and how school as context interacts with play, which is often in ways that undermine the space and opportunities play is given. Based on existing research, the article is critical of the tendency to accord a secondary role to play in school or to instrumentalize play as a didactic tool for learning. The article links to existing play theory, but at the same time develops the concept of play order, through *an ecosystemic understanding*, which makes it possible to look holistically at how play in school can be integrated and provided for. Considering that more and more pedagogues are working in schools and directly involved in teaching, and afterschool clubs (SFO) are increasingly handling schooling tasks, the authors of the article argue that play is worthy of recognition in both practice and theory.

## Introduction

This article is about practical and theoretical challenges when working with play in school. Through the concept of play order we argue for a need to develop an ecosystemic perspective that can aid a nuanced understanding of the connections between play and school in an empirically sensitive way.

In Scandinavia, play is traditionally seen as having intrinsic value for children’s lives (Lillemyr, 2003; Øksnes, 2008, 2019; Ødegaard and Hedegaard, 2020). A large number of research projects attach importance to play in early childhood, i.e., in crèches and kindergartens (Bork and Lund, 2020; Ødegaard and Hedegaard, 2020; Skovbjerg, 2021). Studies of kindergarten children are characterized by their interest in play from a broad pedagogical perspective, as a pedagogical tool for child development, but also as a valuable form of social interaction practiced by children and accorded space and time and supported by the professionals working with the children (Winther-Lindqvist, 2010; Bae, 2012). Still, play in school remains a much-debated topic – among theoreticians as well as practitioners – resulting frequently in play being marginalized and seen as isolated from the school’s core task, just as schooling is thought to have nothing to do with play (Møller et al., 2018; Øksnes and Sundsdal, 2020).

Article 31 of the United Nations’ Convention on the Rights of the Child stipulates that every child has the right to play (UN 2013). Looking at schools, the picture is quite different, and less intrinsic value is attached to play, which is seen more as a didactic tool serving a particular scholastic purpose. There is therefore much to suggest that play is deemed to be valuable for children hence it gradually develops into something more disciplined and becomes subordinated to the core task of schooling once children reach school age (Gilliam and Gulløv, 2012; Jørgensen and Skovbjerg, 2020; Øksnes and Sundsdal, 2020). In relation to existing research, we address three main concerns relating to play in school: First, in schools play is carried out within well-defined boundaries of time and space: during breaks and in the playground. Second, various forms of play are often clearly separated from the academic content and the teaching activities. Third, certain forms of play are not accommodated at all (Møller et al., 2018), and as a consequence, children are seen first and foremost as pupils, and not as children. In the words of Øksnes and Sundsdal (2020), it is characteristic of schools that they “do not see play as essential for the actions of children and young people”, p. 64 (authors translations)]. The projection of concepts associated with play and school also confirms, for example, the fact that the concept of free play is dominant among professionals (Skovbjerg, 2021), and the applied understanding of free play contrasts with the forms of play that can be organized in school (Øksnes and Sundsdal, 2020; Skovbjerg, 2021). Danish political reform stipulates that pedagogues must to a higher extent work in schools and support teachers during classes and teaching. This emphasizes the need to manage both professions; teachers and pedagogues within the purpose of the school (The Danish School Reform, 2014).

The above concerns and research on play in school point to practical as well as theoretical challenges and a need to replace the landscape of unhelpful dichotomies with a holistic perspective through the development of concepts that can aid a nuanced understanding of the interaction between play and schooling in an empirically sensitive way. We achieve this through what we define as an *ecosystemic understanding of play in school* and through specific analysis of play situations, which is based on empirical material from a design-based research project about designing for play in two schools in Denmark. An ecosystemic understanding is defined with inspiration empirically from Kampmann (2009) as an understanding that sees interaction, interrelatedness and holistic orientation as core values for the way we perceive play in school, and where these values guide the pedagogical work of the professionals.

The theoretical and scientific grounding of an *ecosystemic understanding of play in school* is based on a socio-analytical perspective, which is developed by the Danish philosopher Lars-Henrik Schmidt (Schmidt, 1988, 1999). The socio-analytical perspective is a philosophical perspective that is critical toward essentialistic assumptions about what a community is and what a subject is. Instead, it highlights relations and connections as important issues when describing and defining what a community is and how a subject is understood. In order for us to understand community and subject, we have to look for meaning and definitions in connection to the contexts in which communities and subjects exist. The description of meaning must then be closely related to these connections in which meaning takes place. With inspiration from Schmidt and the socio-analytical perspective, we are able to underline that play in school must be seen as a holistic ecology and that the ecosystem is continuously evolving through interactions and interplay.

The purpose of this article is to contribute to this new understanding of the ecosystem by developing the concept of *play order* inspired by Schmidt’s concept of order (Schmidt, 1999) to capture the interaction and interplay going on within this ecosystem. Based on empirical data collected over a 3-year period, the mood perspective (Karoff, 2013; Skovbjerg, 2021), and the concept of *play order*, the article intends to answer the following two research questions: (1) What ecosystemic interactions take place between play and school? (2) How can the concept of play order be developed in relation to those ecosystemic interactions? The concept of play order makes it possible to take an analytical approach to play in school, which is relevant to a field of practice that we have already established is increasingly characterized by close collaboration between pedagogues and teachers, by after-school clubs (SFOs) being closely involved in the schooling of children from preschool to year 3, and by distinct changes to the professional role of pedagogues (Graversen and Ringskou, 2015).

The article’s theoretical contribution of play order will be seen in relation to play research, which in various ways points to play being distinguished by special logics. Huizinga (1938) is interpreted aiming at *the magic circle*, and Bateson (1972) and later Goffman (1986) talk about the *framing* of play. The point made by all the theorists is that play takes place in an imaginary or material space and according to different orders of meaning, which are distinct from the ones known from everyday life (and thus also from life in school). Huizinga, Bateson, and Goffman all advocate the importance of understanding play from the perspective of play, and of acknowledging the existence of frameworks and rules that determine what is meaningful for those engaged in play. At the same time, these orders of meaning are separate from everyday reality and must be viewed as such. The concept of play order is aligned with this understanding, but at the same time it generates a possibility for analyzing the special play orders, which cannot be separated from what goes on in school, but actually interrelate and interact with schooling. Thus the concept of play order intends to thematize interaction and interrelatedness which are continuously happening, rather than categorical and defined separateness and marginalization. The paper presents new knowledge about play in school, and through theoretical development, the paper takes the role of play seriously, hereby enhancing an uncovered research field. In relation to the special issue *Breaking, making, learning: Analyzing Processes of Play* the paper contributes with a novel understanding of the dual nature of play processes in school contexts. Furthermore, an underlying argument is that a sensitive understanding of children’s play in school is necessary in order to support children’s social practices. Furthermore, we see a vague thematization of play in the current journal, which is why we believe the theoretical and conceptual development of this paper is a valuable contribution, which can help develop the field in future research.

We begin with a presentation of *play order* to unfold the theoretical framework of the article. Next, we present the method and the research context of the analyses. The third section contains empirical examples, which we analyze through the lens of the presented theoretical framework. The article concludes with a discussion of the implications for both practice and the theorization of play in schools based on an ecosystemic understanding.

## Theoretical Framework: Play Order

The article’s theoretical perspective on play is based on the mood perspective (Karoff, 2013; Skovbjerg, 2021). The mood perspective offers a common human perception of play as a way of being that is not confined to childhood, but which is a form of social interaction that we as humans engage in throughout our lives. As stated in Skovbjerg (2021): “Play tells us something basic about what it is to be human, and games have taken place throughout all of history and in all human societies. It is just something we do, and unusually it seems like child’s play” (p. 9).” The inspiration for the mood perspective is partly drawn from the international play research that sees play as a special form of social interaction whereby you become part of a mood (Sutton-Smith, 2001; Henricks, 2015). In addition, the mood perspective is inspired by Scandinavian childhood research, for example, the research by Flemming Mouritsen (1996), who in the late 1980s stressed the importance of the participants’ own commitment, ideas, interests and life situation when seeking to understand play and not reducing play to a function of learning. Mouritsen’s thinking initiated a field of research, which has come to include a large number of prominent Scandinavian figures defining play research as an independent field of research exploring an important aspect of children’s lives (Øksnes, 2019; Jørgensen and Skovbjerg, 2020; Øksnes and Sundsdal, 2020). Play is a challenging theoretical phenomenon because it is difficult to adopt and rely on a single analytical perspective. In the words of Sutton-Smith in *The Ambiguity of Play*: “We all know what playing feels like, but when it comes to making theoretical statements about play, we fall into silliness” (Sutton-Smith, 2001, p. 1). The statement highlights the fact that we can all relate to what playing feels like, but may have a harder time coming up with a language suitable for conveying an understanding of play as a phenomenon. For the same reason, there is a need to develop a language for play in school that is relevant and productive to research and pedagogical practice. In connection with the mood perspective on play, a number of theoretical concepts have been developed (Karoff, 2013; Skovbjerg, 2021), which can generate a fundament for exploring play from a theoretical and empirical point of view. In this article, we work specifically with the concept of *play order* to understand how the interaction between play and school can be understood and conceptualized in the context of the school.

### Play Order

The concept of play order is defined with reference to the concept of *order* developed by the Danish philosopher Lars-Henrik Schmidt (1999), about which Schmidt emphasizes: “In the following, we call the generalized *presupposed purpose* for “order.” Order is what is presupposed and what is sought, a draft or a plan sought to be realized” (Schmidt, 1988, p. 35). According to Schmidt, order is a logic on which actions (practice) are based, and which those involved (in this case) in a play activity have an idea about and which they seek to realize through their play actions without necessarily being able to define exactly what is going to happen. So it is through practice that the order emerges, while at the same time setting the direction for actions. Following Schmidt’s definition of order, the term play order can be applied when aiming at the connectedness and relatedness that play has to the context in which it takes place. The basic assumption from the socio-analytical perspective is that the description of play can only be made in relation to a certain background of meaning. But at the same time, that background cannot be understood as a final reference for what is essential. In that sense what is meaningful in play does not exist as something which needs to be revealed. Instead, meaning emerges and does not originate elsewhere but from the interactions and practices within the play order.

Play must *consistently* relate to some sort of order to confirm what the order is and to confirm the meaning of that specific order, and this happens through play actions. The important aspects of a play activity are accorded value. In the empirical material presented later, for example, children engaged in role-play are found to remind each other that: “then we said that,” after which they continue playing. As a kind of affirmation that they are in agreement that something has value. Thus, not all actions are accorded value in a play order, but the participants can only test this *through their doings*. The play order must be seen as the organization of actions, materials, and relationships practiced by the participants, and value is determined through continuous maintenance and repetition. On the one hand, the play order is the prerequisite for a play activity to happen at all, and on the other hand, the play order is under continuous construction. This does not mean that the play order should be seen as predefined instructions for action to be adhered to by all. Rather, the point in this context is that the play order – through the doings of the participants – is in constant flux between *affirmation*, *development*, and e*xecution*. The children establish the play order through their doings but where the important thing is that they do so without trading (Schmidt, 1988, p. 58), i.e., they do not necessarily openly negotiate, but move forward and establish and maintain the play order through their doings. Through the relations that occur within the play order, the order makes sense to the participants, without them referring to an external well-defined idea about play order. The participants do what they do, and the play order comes to life. As will be visible in the analyses, disagreements about the play order may arise. This happens in situations where the participants thought they had agreed on something, but in fact they disagree. Skovbjerg (2021) argues: “Play breaks down when disagreements become insurmountable. It also becomes clear that the mood they thought they shared, they didn’t agree on after all (p. 89).” The play order is therefore not necessarily characterized by harmony and friendship, but may also be characterized by struggle and conflict. In this way, participation in a play activity is a constant and demanding balancing act between taking part in and contributing to a play order and then abandoning it. In the words (Skovbjerg, 2021): “Play orders that become too rigid or unpredictable quickly lose their relevance, in the same way that play orders in a state of constant quiver and at risk of collapse quickly prove exhausting and thus cease to be meaningful Skovbjerg (2021, p. 41).”

## Research Context and Methodology

This paper is based on large-scale empirical fieldwork undertaken in connection with the research project entitled *Can I join in? - Play, Inclusion and Communities in School*. The project runs from 2018 to 2022 and is funded by *Danish Independent Research Foundation.* The project involves 600 children, 40 pedagogues, 2 schools, 7 Danish researchers, and 2 international researchers. In The Danish School Reform (2014) required pedagogues to work with children in the school context and also collaborate together with teachers, also in relation to school requirements, which are new to them, but also to hold on to main content of pedagogue relational work. This change in pedagogue’s workspace has therefore reinforced the need to focus on how pedagogues design for play in the school context. Therefore, pedagogues and children are of primarily practical and analytical interest in this project. The focus on, collaboration with and ongoing dialogues and reflections with pedagogues has generated strong confidentiality between the pedagogues and involved researchers.

The research methodology is based on design-based research (DBR; Brown, 1992; Barab and Squire, 2004; Anderson and Shattuck, 2012; Ørngreen, 2015; Ejsing-Duun and Skovbjerg, 2018; McKenney and Reeves, 2018; Jørgensen et al., 2021), action research (Rönnerman and Salo, 2012) and ethnographic interventions and experiments (Akama et al., 2018; Criado and Estalella, 2018). The overall purpose of using this methodology is to be able to research and develop playful processes in collaboration with pedagogues by using design processes. The design-based research project focused on the pedagogues’ perspective and their collaboration with children. The use of ethnography methods generated knowledge about sensory and visual dimensions, which are part of design processes. The design phase with the two schools was carried out during a period of 1, 5 years and resulted in 56 design experiments. The design experiments (hereinafter referred to as designs for play) took place both as part of the school’s planned supportive teaching activities, which aim to “support a varied school day with a mix of teaching activities, etc., […] or which aim to support the receptiveness to teaching, social competences, all-round personal development, motivation, and well-being of the pupils” (Section 16a of the Danish Folkeskole (Consolidation) Act (*Folkeskoleloven*)), and in the after-school club (SFO) in the afternoon. The purpose of designing for play is not to decide *what should be played*, but to establish a framework for varied and experimental conditions for *playing*, i.e., for play orders. The design phase was organized with reference to a model developed on the basis of a design-based research approach and inspired by Barab and Squire (2004), Gynther (2011), Jørgensen et al. (2021). The model consists of four domains, which draw upon specific research practices and paradigms. The four domains are; (1) Context – the problem is settled. (2) Lab – principles for the experiments are constructed collaboratively. (3) Experiments – interventions with design experiments with the empirical field. (4) Reflections – reflections on insights and experiences from the design experiments and discussion of possibilities of exploring further development of theory, principles and designs. The 56 design experiments made by pedagogues were thematized with a specific type of play, inspired by Huges: Creative Play, Role Play, Dramatic Play, Construction Play, and Movement Play. Each play type was enrolled in a 6-week design phase, which consisted of interactive processes between reflections and experiments.

### Empirical Material and Analysis

The empirical material generated during the design-based project consists of field notes, videos using IRIS, ethnographic videos, and photographs (Pink, 2007, 2011; Sand et al., 2021), audio recordings of play situations, interviews with children and pedagogues. Furthermore, several conversations at the reflection meetings have been recorded and analyzed as empirical material. The production of data was fully GDPR-compliant. Since the children were between six and nine of age, written consent was obtained from the children’s parents in a manner consistent with the Danish Code of Conduct for Research Integrity. Written content to use visual material is also given by leaders, pedagogues and parents. The name of children, pedagogues and schools are furthermore anonymized. In the research team, we used the digital data processing program Dedoose which enables open and focused empirical coding as well as memos (Cgarmaz, 2006). The open coding revealed a pattern of codes related to school as a context for play, for example: Rules, regulations, time schedule, time structure, learning, separate offices for pedagogues and teachers, pedagogues’ insecurity about working in school, messiness, materiality, moving bodies etc. Within the research team, we held regular data workshops as well as visual exploratory workshops where empirical examples were unfolded and discussed.

Since the two schools were affected by Covid-19 regulations during the period of the design experiments, we had continuous dialogues dealing with ethics and how we could reduce demands or somehow align our involvement with the schools to their everyday practices. This type of ethics is described as situational ethics (Hammersley and Atkinson, 1983) or ethics in practice which points to how researchers make judgment and decisions in real-time through careful observations, awareness and sensitivity (Van Mechelen et al., 2020).

The analysis strategy applied in the article is inspired by grounded theory (Cgarmaz, 2006; Salmona et al., 2020) and consists of a combination of empirical analyses of play situations and the concept of play order. It is possible through the design experiments and the production of empirical material to gain insight into the emergence, maintenance, development, and settlement of play orders, and the school’s interaction with these. The analysis is divided into three parts based on repeated readings and discussions of the empirical data, revealing that different types of interactions and frictions characterize play orders in schools. The concept of play order underlies the analysis of concrete conditions for play in school and shows how the interaction of school and play is characterized by both clashes and divergence while giving rise to frictions both ways.

## Analysis: Play Order in School Space

The following analysis consists of three empirical analyses, thematizing play in school: (1) how the play order is established in schools; (2) when the play order meets the logic of school, and (3) when play orders involve conflicts and sensitive interactions.

### When Play Order Is Established in School

The first highlight looks at the way play order is established in interactions between children, pedagogues, materials and spaces in the school. We start with an excerpt from the field notes:

*Iben, a pedagogue, has announced that a café pretend play activity will start in the classroom during after-school club (SFO) hours. Children from the preschool year are running around asking “When does it start?” The pedagogue arrives and asks the children to help get out the kitchen things. Several children run along with her. Soon after, they come back carrying groceries, a cash register, shopping baskets, and kitchen utensils. As soon as everything is there, the children start handing out shopping baskets, and the pedagogue positions the cash register on a small bookcase. The kids jump up and down, shouting: “I want to be the beeper,” “I’m the shopkeeper,” “I’m a waiter,” “I do the sorting.” The children run around stocking the shelves, or putting groceries in their baskets. One of them is manning the cash register and opens and closes it over and over again – thus making constant CLING and SWISCH sounds. Muddie, who is walking around with a basket, suddenly slumps down and starts limping: “I’m an old lady.”* (Fieldnotes, 11.11.2019).

The empirical example shows that the children are excited about what is going to happen and are eager to begin playing. As soon as the pedagogue brings along the materials, the children begin exploring and finding ways to start playing. They do this by seizing hold of the materials made available to them, which are the groceries from the SFO’s toy box. The children use their prior knowledge of the material and theme to start imagining how they can play. The play design is role-play, more precisely café pretended play, and the material made available to the children causes them to suggest possible roles, which match that particular type of role-play. Through these practices, through the play thematics and materials, the children orient themselves into a context of meaning that is meaningful to them, and they start engaging in practices relevant to that context: One of the children operates the cash register, another walks around with a shopping basket. The play order is being established through the immediate logic recognized by the children based on the materials, thematics and possible roles. Initially, the children lead the wayfinding roles like “shopkeeper” and “waiter,” and these roles at the same time point to certain actions performed by the roles, and which the children can start to take on. In addition, Muddie makes an active contribution to the play order based on his knowledge and ideas about old ladies to bodily support the play order that is in the process of establishing itself. He stops and starts limping around, pretending that his body is old. The establishment of the play order is further supported by the pedagogue in the following empirical example:

*The pedagogue asks: “I wonder how you actually get something to eat in this restaurant?.” She then calls out: “Waiter!” […] Muddie, who was playing an old lady, is now busy building a burger. He comes over and serves the burger to Iben. He says there’s ketchup in it. Iben pretends to throw up. Muddie laughs and returns to the burger-building station while pretending to throw up. Silje comes over to ask if Iben would like some yogurt. Iben says yes pleaseif it comes with pineapple. Muddie says he is going to make a spit cake. A couple of children come along with a bag of Monopoly money. One of them says that now she wants to buy a restaurant ticket with all the money…”* (Fieldnotes, 11.11.2019).

The play order develops procedural through the café theme, which is evident, for example, in the way that the café becomes a restaurant with waiters. At the same time, the children seize ideas that emerge along the way and contribute to the play order. This means that the children get ideas along the way while incorporating the materials that have been made available to them, including plastic play food, the bag of Monopoly money, etc.

A number of children contribute to maintaining the play order. Muddie continues to explore the play order by experimenting with different types of pretend play. He accepts the framing of the play order through bodily acts, and actively contributes to the play order by going along with pretending to be sick. He trustingly adds actions to the play order, the unfolding of which proves meaningful within the play order, such as stirring ketchup into the coffee and making spit cakes. More children come along and start running back and forth between the cash register, restaurant tables and the kitchen. Then the following happens:

“*Iben now appears with a pile of tea towels and aprons. “The chefs have to wear aprons,” she shouts. “The waiters must carry a tea towel draped over their arm*.” *“I want to do that!” several children shout. A lot of children have joined in. Iben demonstrates how the waiters should carry the tea towel, then she says: “Shouldn’t we have a children’s play corner? Just in case families with children come to the restaurant.” Some of the children set to work straightaway building a children’s play corner. The other children get help tying their aprons. Jonas struts around with a tea towel nonchalantly draped over his arm. “Who wants to come along and announce that the restaurant is open?” asks Iben. A couple of children say yes. They then march off to the cloakroom together, shouting in unison: “THE RESTAURANT IS OPEN.” Several times*.” (Fieldnotes, 11.11.2019).

The empirical material is exemplary in illustrating how play can take place within a school context, through the constructive interplay between pedagogues and children. The pedagogue Iben continuously supports the establishment and affirmation of the play order by handing out tea towels and aprons. By providing new materials, she makes it possible for more children to join the play order. Finally, Iben supports the creation of a shared mood by gathering together a small group of children who proceed to march through the cloakroom, shouting and singing. The children accept Iben’s invitation to add to the play order when she asks whether they should have a children’s play corner in the restaurant. Overall, the play order is established organically through constitutive interactions between the play universe, the actions and doings of the participants, the available materials and the verbal expressions exchanged by the children and the pedagogue. The pedagogue makes suggestions, which are seized by the children, who also respond by coming up with ways of further supporting the play order. Together the children and the pedagogue establish the play order, while at the same time constantly keeping the play activity going by affirming the play order. Thus, the example illustrates how they are sensitively attuned toward each other, their surroundings and the materials they have been given. Their contribution to the play situation is aligned and hereby they confirm what is meaningful for them about the play order, and thus also what is not. What is meaningful is not predefined, but it becomes meaningful as the situation unfolds. In other words, the connected interplays make up what we can refer to as an ecosystem.

### When the Play Order Meets the Logic of School in the Form of Materials, Physical Spaces, and Rules

In the first part of the analysis, we saw how play could be established in school, generated by the children’s ideas and the support of the pedagogue in a situation where the primary purpose of the play order was play. In the following empirical material, the interactions between play order and school logic become a bit more complicated. The empirical data includes several examples of transitions that are neither smooth nor easy, but rather made up of frictions between play orders and school logic as regards expectations concerning the school’s physical spaces and rules. We will look more closely at this in the next example:

“*Sofie, a pedagogue, has started picking up money and says: “Otherwise the coins will be swept up and thrown out, and then they will not be there to play with tomorrow.*” *She stops picking up money and walks over to the laminator and starts laminating signs. Mette, a teacher, walks in. She starts to move a table and some chairs into the middle of the room, where the children were just romping around. “You simply have to start clearing up now,” says Mette. She is standing in the middle of the room. Her voice is low, but sometimes rises. She shuffles her feet impatiently. Tilts her head ever so slightly. Sucks in her lips. Her chin quivers. She tells Sofie a parents meeting is due to start shortly. Sofie addresses the children and says that in 5 min they have to start clearing up. Mette, the teacher, says (with a little laugh): “But they’re already here.” Sofie looks at her watch and calmly continues laminating. “Bertram, you simply must put those chairs up now,” says Mette. “You simply must finish now.” She then raises her voice: “NO,” quickly lowering her voice again: “Quite frankly, I’ve just put those chairs down, they’re needed for the parents meeting.” She walks round among tables. “You simply need to finish what you’re doing now,” says Mette. “You have to get out of here.”* (Fieldnotes, 19.10.2019).

The pedagogue is aware that the play order involves clearing up, and she is aware that if she does not look after the play materials, then they will be thrown out. Not only is there a risk that the play materials will be thrown out, in this situation the teacher’s plans for using the physical space for a different activity are also being challenged. In this instance, the play order is marginalized and isolated, and it is eventually terminated because the school logic takes precedence. The play activity has to stop – right now – because Mette has a parents meeting coming up. In the example, the teacher, Mette, shows a lack of sensitivity to the play order and the values that the children attach to their activities. The play and school logics seize to co-exist and instead they collide when there is no mutual understanding between the pedagogue and the teacher.

In the empirical data, there are also several examples of transitions between play orders and school logics that result in the play orders and school logics becoming interwoven. In the following example, the play design is creative play, and the pedagogues defined the play activity Quicksand:

*“The children are gathering large piles of mud with their hands, then they pick it up and throw it into the canal. A girl is merrily swinging a filled watering can around. Spilling a bit here and there and also in my notebook. The boy Henrik picks up a piece of wood and holds it in his hands. “Time to go insiiiiiide,” shouts the pedagogue. The children carry on playing with the water in the canals and show no inclination to stop. “We’re going inside now,” the pedagogue repeats. The bell rings. Some of the kids start going inside. I walk alongside the boy Henrik. He is still holding the piece of wood. He brings it into the cloakroom, where a teacher, Jesper, says hello to him. Henrik shows the piece of wood to Jesper and starts telling him what he has just been doing”* (Fieldnotes, 13.09.2019).

Henrik has just been deeply involved in the play order in the school playground together with his friends. They have played gold digger, and as can be seen from the field notes, the pedagogue has to tell the children several times that it is time to go inside because the bell rings. Henrik takes his piece of wood with him as he goes inside. He holds on to it, and continues fidgeting with it and picking out fragments of wood. He sits down in the cloakroom with the piece of wood next to him. When he meets the teacher, he tells him what he has just been doing and that he would like to continue playing even though it is time for a lesson. The following observations are made:

*“Jesper listens to Henrik’s story and is keen to know what Henrik has been doing outside. Henrik hangs up his outerwear in the cloakroom, and as he makes his way to the classroom together with Jesper, he continues to hold on to the piece of wood, picking small bits off it and talking about water and mud. As he enters the classroom, the teacher Jesper asks Henrik to put the piece of wood on the windowsill as it is time for them to do something else.”* (Fieldnotes, 13.09.2019).

Henrik draws his play experience into the school building, and by having a conversation with the teacher who acknowledges that a rapid shift from one play order to a school logic can be difficult to handle, Henrik is able to successfully transition from the play order to the school logic without experiencing a complete disruption - being aware of the fact that some children can experience a rapid shift from one play order to a school logic being difficult to handle. Eventually, Henrik has to put the piece of wood on the windowsill, but he accepts this because he understands that now something else has to happen.

During our fieldwork, the children and pedagogues repeatedly cite having to clear up play materials and that a consequence of play materials being thrown is that play that occurs over long periods of time are difficult to set up. The materials associated with the play order will be thrown out by the teachers if they are not cleared away by the children or the pedagogues. In the words of one of the children: “*It’s always like that*,” (…) “*She also scolds us for leaving our ice lolly stick houses on the windowsill*. *She says it’s messy and untidy.”* The situations suggest that the physical spaces and the materials are first and foremost expected to align with the school logic, with little sensitivity for play orders with different values and different orders of meaning. When masks used for a theatrical play activity are suddenly thrown out, or a cave is dismantled by the janitor or everything has been cleared away to make way for next time the cleaners come, these are examples that illustrate how, in schools, the school’s logic dominates the way play is practiced. At the same time, it reinforces the dichotomy between school and play, where children experience that play is for breaks or after school (Øksnes and Sundsdal, 2020). Since pedagogues to a larger extent work in schools in Denmark, where they have to collaborate with teachers, the ecological perspective can help nuance the connections, possibilities and frictions temporally, materially and physically. The ecosystemic perspective helps us to see the conditions for making holistic connections between play order and school logic. This does not mean making equal space or structures for play to occur, but as the example with Henrik illustrates, to make holistic transitions between play activities and school-oriented activities.

### When Play Orders Involve Conflicts and Sensitive Interactions

In the previous part of the analysis, we saw how play orders clash with school logic, and how the clashes give rise to frictions temporally, materially and physically and in relation to rules. In the following we will examine two empirical examples that show how play orders characterized by strife and conflicts can lead to friction in relation to the school logic.

The example below relates to movement play, and the play activity designed by the pedagogues is called Nerf Gun War. The example shows how the play is framed by the pedagogues in a way that controls and steers clear of conflictual and aggressive play.

*“The children have been playing Nerf Gun War for 90 min. In the end, they change the rules. One of the pedagogues, Mikkel, says that from now on everyone can die. All the children must pick up six bullets and get ready. If you die, you’re out. The team where everyone dies first loses. The whistle is blown. The way they move changes. Everything is more intense. They creep around. They do not just stand up. They go for stealth attacks. One boy walks to the front line and attacks the others. But is met by a girl pointing her gun at him from the other side of the wall. Shocked, he quickly stands up and runs back to the high wall behind which the others are hiding. “It’s too risky,” he says.*

*The children are hit one after the other. They’re out. As fewer and fewer children are left playing, something new happens: They start using more language. They start wrangling about who shot whom, and whether some people are cheating.*

*“You’re cheating.” “No, I’m not.” “Yes, you are.”*

*The boys speak loudly. They shout.*

*Mikkel comes over to me and says, “Do you see what’s happening? 5 min, then they start arguing. I thought you should see it. Happens every time.”*

*The whistle is blown for the last time. The boys carry on bickering.”* (Fieldnotes, 22.11.2020).

The above empirical example illustrates that the boys start fighting when the play order is changed to include a competitive element. The competitive element is part of the play order practiced by the children. The pedagogues have mentioned several times that they try to avoid the competitive element in their play activities, both because it excludes some children from participating and because it gives rise to internal strife, particularly among the boys. However, the competitive element not only leads to strife, but also to another bodily way of playing, which is seen in the way the children creep around in a very engaged way while taking certain risks, and generally from the more intense atmosphere of the play situation. However, it would seem that this aspect of the play order is not articulated by the pedagogues, who focus instead on the fact that the boys start arguing with each other. The empirical example indicates that within a school context it is difficult for the pedagogues to accommodate the strife that is part of the play order, which might be related to a worry that the children may get hurt, to certain expectations about the way you behave toward others and also to how the school as a context have certain rules about rough play, loud noises, etc.

The following example shows how four boys sensitively succeed in adapting their play order to a classroom setting, rather than raising awareness of a play activity that could be perceived as aggressive and conflict-ridden. The play design is creative play, and the play activity is called Word Relay:

“*The groups work with small materials such as pipe cleaners, matches, Plus-Plus shapes, etc. All the children are busy playing at the tables and on the floor in the classroom. A play activity has evolved among four boys. They have built small firearms out of Plus-Plus shapes, and start shooting each other. They roam the width and length of the classroom. They run around, duck down, arch their backs and exclaim if they are shot. The creative play activity has evolved into a role-play activity for the boys. One boy is standing next to a chest of drawers, he raises his hands, shoots, makes a quiet hiss shooting noise, while his hands pull the trigger twice. He straightens his head and starts running, tip-toeing slightly. Muddie is under a table. He is holding a machine gun and in a whispering shooting voice he sends off a series of bullets in quick succession “deu, deu, deu, deu,” rolling his tongue.*

*They run past me, but don’t see me, see only each other. The fact that they are keeping their voices down probably explains why they are not asked to stop. Two other boys go over to Muddie. A boy shows off his gun, the others gaze at it, mouths and eyes wide open. The battle continues. They run past me, shoot, run over to the door. One boy is caught in a corner, and they move from guns to body contact. With his leg bent at the knee, Muddie makes cautious kicks against the other boy’s stomach. The boy is bending over, his back against the wall. He manages to flee the corner. They continue running around the room*.” (Fieldnotes, 04.10.2019).

The boys’ fight is silent, no one notices them. But they have created their own play space within the play activity developed and framed by the pedagogues. The empirical material shows how the boys sensitively decode the classroom and the play order in progress, and adapt their play activity accordingly. This is a condition for them to be able to play their own game.

Within this section we have analyzed two empirical examples where pedagogues in one situation explicitly try to frame a play activity in a way that avoids, controls, and steers clear of conflict and potential aggressive play. In the other situation we see how boys play a potentially loud and conflictual play activity, but they do it with a sensitivity toward the surrounding space, people and logic. The examples tell us that generally the school can accommodate play, but that the degree to which play can be accommodated depends on the way in which children manage to be sensitive to the temporal, spatial and relational dimensions. But where does that leave children who play with less sensitivity? The following discussion will circle around questions of this nature.

## Discussion: Play Order as a New Understanding of a Perspective on Play in School?

The starting point for the article is that play in school is a dichotomous field of study, and the article contributes a new understanding of and perspective on play in school, which is an ecosystemic perspective, that sees interaction, interrelatedness and holistic orientation as key to understanding and working with play in this context. Referring to longitudinal ethnographic studies of Danish institutions an organic understanding is essential in order to work constructively with some of the underlying assumptions and practices related to play and children’s behavior. Hence, the school’s agenda may tend to order and direct the way children and adults behave and interact. Drawing on the work of argue that Danish institutions are based on an ideal of civilized upbringing, and that the behavior of many teachers and pedagogues is determined by this ideal in their everyday engagement with the children. They argue:

*“Boundaries are culturally relative markers of distinctions between behavior, values and people, in relation to this, children who are* ***too pushy***, *children who* ***invade other people’s spaces***, *who* ***know no shame***, *and who thus, in various ways, do not accept other people’s boundaries, or who may even be* ***boundless***, *will be regarded as uncivilized.”* (Gilliam and Gulløv, 2012, p. 261, original highlight, own translation).

The highlighted terms are empirical statements from Gilliam and Gulløv’s studies and provide insight into the existence in Danish welfare institutions of markers that, for example, have to do with the ability to self-regulate, maintain order, be quiet and the setting of boundaries. When talking about play in schools, where the agenda is largely guided by learning goals, and based on the empirical analysis of this paper, we find it necessary to reflect on the interrelationship between play order and the ideals of civilization highlighted by Gilliam and Gulløv. How is play valued if it is wild, loud, conflictual, relies on materials not having to be cleared away, unfolds over a period of time, and transgresses the generally applied markers of civilized social practice?

Furthermore, civilized social practice is according to parents, pedagogues, and teachers connected to being good at playing. Gilliam and Gulløv explain:

*“It seems to involve an ability to invent what to play, but also to be able to integrate other children and their ideas in the development of a common, inclusive togetherness. (…) If we take a closer look at the importance of* ***being good at the social***, *the term refers precisely to the child who manages to include others in play and talk, who engages in positive relationships with others and plays across the group of children. Negative formulations of the type:* ***he does not know how to engage with the social community***, *tells us that the community is not just a goal, but also a measure”* (Gilliam and Gulløv, 2012: 266, original highlight, own translation).

According to Gilliam and Gulløv, play is a valued competence in school settings and at the same time, it is connected to certain social abilities as: being inventive and creative, integrating other children in play, taking other children’s ideas into account, being empathic, following one’s own needs while being able to listen and not suppress others, and finally playing with different groups of children. Based on many years of observing children play, we see a variation of how children engage in play and how children find it meaningful to participate socially through play. Therefore, we find it problematic to operate with such defined norms for what good social play practice is. What does it mean when Gilliam and Gulløv state that the community is not just a goal, but also a measure? It means that civilizing processes define standards for behavior and entail that some practices and people are recognized and some are not (Gilliam and Gulløv, 2012). When it comes to play, it is problematic since playful practices have multiple expressions, forms and intentions and because play to many children is a practice that can allow them to participate socially in ways, they find meaningful (Skovbjerg, 2021).

The conditions for play in school are also related to organizational and structural dimensions, and the ecosystemic understanding raises questions of how to approach play in school holistically, specifically looking at: Why is there a need to sharply demarcate the management of pedagogues and teachers? Why do different groups of professionals have separate offices and coffee rooms? Why is the school day split into time for playing and time for learning? Why are some materials intended for learning situations and others reserved for play? And why are materials organized according to values such as tidiness and order? All these questions call for a basic discussion of how – in the light of an ecosystemic framework of understanding – we would like the interaction between playing and schooling to guide the professionals’ work with children’s play in school.

In our analyses of the play order concept, we have shown that it allows us to conceptualize and theorize play in school in a way that sheds more light on the nature of relationships and connections, while at the same time capturing frictions and disputes. In this perspective, we highlight the importance of moving away from concepts such as “free play” and the idea that institutionalized play is not real play. Such an approach creates conceptual gaps that are problematic when wanting to capture the nuances and collaborative possibilities of play, and at the same time makes it impossible to understand play in school, which in turn complicates pedagogical decisions related to play.

The intention is not to focus on harmony or disharmony, but to clarify the diverse interactions and frictions associated with play in school, more specifically: to generate transitions between play and teaching, understand play as a practice in its own right, that school staff include each other’s perspective and the logics they are surrounded by in their workday, to reflect about the tendency to control noise, unrest, clutter, etc., since it leads to a tendency to overlook other essential parts of play. And furthermore, to examine how play and school logic can inform each other.

Nordic research, in particular, tends to conceptualize play as free play, and the argument for moving away from such conceptualization in the school context is twofold: Discursively, play first and foremost comes to mean adult-free play, and secondly, research shows that it deprives the professionals of the possibility of intervention or involvement when play is something that happens outside the adults’ space for action. These positions are reiterated, for example, by Øksnes and Sundsdal (2020), as they emphasize the importance of children having their *own* play space, and from that position they stress that this play space is different from a play space populated by adults. At the same time, they point to the need for adults to show special pedagogical tact and to handle play situations gently. The point here is that an ecosystemic understanding of and perspective on play in school can help conceptualize pedagogical tact and sensitivity and make it possible for it to be exercised. If we go along with Gilliam and Gulløv, this could mean that the school as a context would not only lead to certain pupil positions and certain forms of practice, but would be able to accommodate playful children in an acceptable way.

Further conceptual development and practical research will be needed going forward to ensure that play in school is not marginalized; as Møller et al. (2018) point out, there is a need for play in school to be taken seriously: “Play takes hard work and dedication, on the part both of those who are playing and the professionals wanting to create frameworks and conditions for play. The fun only really starts when it gets serious” [Møller et al., 2018, p. 9 (authors translation)]. Following this argument, we argue that an ecosystemic perspective can help create harmonic conditions for play in school contexts. At the same time it requires an awareness and willingness to have a dialogue between pedagogues and teachers about how play order and school logics can constructively generate space and even support each other. The reflections call for further research exploring (and designing for) play in school contexts.

## Conclusion

This article concerning play in school points to practical as well as theoretical challenges and a need to replace the landscape of unhelpful dichotomies between play and school with a holistic perspective that can aid a nuanced understanding of the connections between play and school in an empirically sensitive way. This article develops an ecosystemic understanding of play in school through the concept of play order, which makes it possible to look holistically at how play in school can be integrated and provided for. Through empirical analyses, the article shows how play in school is established and maintained, and how the school as a context interacts with play. The concept of play order thus intends to thematize interaction and interrelatedness rather than separateness and marginalization and hereby shed more nuanced light on interactions and frictions associated with play in school. For example: generating transitions and fruitful collaborations between play and teaching, that school staff (teachers and pedagogues) include each other’s perspective and the logics they are surrounded by in their workday and generate a reflection about what types of play are usually welcome in school contexts. A first step toward supporting play is to be sensitive to the play order and try to understand play on its own terms. This way of ordering and making play acceptable challenges the school logics in various ways, and the flexible and unpredictable aspects of play makes it difficult for play orders to develop a voice, but it also makes it difficult to prevent the school logics from steamrollering and subordinating play. The ecosystemic perspective helps to clarify how different logics are at stake and how they implicitly or explicitly influence the way in which play in school is practiced. We encourage scholars to take the intersection between play and school seriously and interrogate and experiment with the way in which pedagogues as well as teachers work professionally with play.

## Data Availability Statement

The raw data supporting the conclusions of this article will be made available by the authors, without undue reservation.

## Ethics Statement

Ethical review and approval was not required for the study on human participants in accordance with the local legislation and institutional requirements. Written informed consent to participate in this study was provided by the participants’ legal guardian/next of kin.

## Author Contributions

Both authors listed have made a substantial, direct, and intellectual contribution to the work, and approved it for publication.

## Conflict of Interest

The authors declare that the research was conducted in the absence of any commercial or financial relationships that could be construed as a potential conflict of interest.

## Publisher’s Note

All claims expressed in this article are solely those of the authors and do not necessarily represent those of their affiliated organizations, or those of the publisher, the editors and the reviewers. Any product that may be evaluated in this article, or claim that may be made by its manufacturer, is not guaranteed or endorsed by the publisher.
